# Association of Interparental Violence and Maternal Depression With Depression Among Adolescents at the Population and Individual Level

**DOI:** 10.1001/jamanetworkopen.2023.1175

**Published:** 2023-03-01

**Authors:** Dawid Gondek, Laura D. Howe, Ruth Gilbert, Gene Feder, Emma Howarth, Jessica Deighton, Rebecca E. Lacey

**Affiliations:** 1 University College London Great Ormond Street Institute of Child Health, London, United Kingdom; 2 Department of Population Health Sciences, University of Bristol, Bristol, United Kingdom; 3 Medical Research Council Integrative Epidemiology Unit, University of Bristol, Bristol, United Kingdom; 4 Centre for Academic Primary Care, Department of Population Health Sciences, University of Bristol, Bristol, United Kingdom; 5 School of Psychology, University of East London, London, United Kingdom; 6 Evidence Based Practice Unit, University College London & Anna Freud National Centre for Children and Families, Clinical, Educational and Health Psychology, London, United Kingdom; 7 Research Department of Epidemiology and Public Health, University College London, London, United Kingdom

## Abstract

**Importance:**

Parental intimate partner violence (IPV) and maternal depression are associated with increased risk of depression in children at the population level. However, it is not known whether having information about these experiences can accurately identify individual children at higher risk of depression.

**Objective:**

To examine the extent to which experiencing parental IPV and/or maternal depression before age 12 years is associated with depression at age 18 years at the population and individual level.

**Design, Setting, and Participants:**

This cohort study used data from the Avon Longitudinal Study of Parents and Children, a UK population-based birth cohort, which initially recruited pregnant mothers with estimated due dates in 1991 and 1992. Data used in this study were collected from 1991 to 2009. Data analysis was performed from February to March 2022.

**Exposures:**

Mother-reported parental IPV was assessed on 8 occasions (child age, 1-11 years). Maternal depression was assessed via the Edinburgh Postnatal Depression Scale or by the mother taking medication for depression, as reported by the mother on 8 occasions (child age, 2-12 years).

**Main Outcomes and Measures:**

Depressive symptoms were measured with the Short Mood and Feelings Questionnaire (SMFQ) and Clinical Interview Schedule–Revised (CIS-R) when the child was aged 18 years. Binary indicators of a case of depression were derived the cutoff point of 11 points or above for the SMFQ and 12 points or above for the CIS-R.

**Results:**

The study included 5029 children (2862 girls [56.9%]; 2167 boys [43.1%]) with a measure of depressive symptoms at age 18 years. IPV only was associated with a 24% (adjusted risk ratio, 1.24; 95% CI, 0.97-1.59) higher risk of depression at age 18 years, exposure to maternal depression only was associated with a 35% (adjusted risk ratio, 1.35; 95% CI, 1.11-1.64) higher risk, and exposure to both IPV and maternal depression was associated with a 68% (adjusted risk ratio, 1.68; 95% CI, 1.34-2.10) higher risk. At the individual level, the area under the receiver operating characteristic curve was 0.58 (95% CI, 0.55-0.60) for depression according to the SMFQ and 0.59 (95% CI, 0.55-0.62) for the CIS-R, indicating a 58% to 59% probability (ie, 8%-9% above chance) that a random participant with depression at age 18 years had been exposed to IPV and/or maternal depression compared with a random participant who did not have depression.

**Conclusions and Relevance:**

In this cohort study, parental IPV and maternal depression were associated with depression in adolescence at the population level. However, estimation of an individual developing depression in adolescence based only on information about IPV or maternal depression is poor. Screening children for maternal depression and IPV to target interventions to prevent adolescent depression will fail to identify many children who might benefit and may unnecessarily target many others who do not develop depression.

## Key Points

**Question:**

Is exposure to parental intimate partner violence (IPV) and/or maternal depression during childhood associated with depression at age 18 years at the population and individual level?

**Findings:**

In this cohort study of 5029 children born in 1991 to 1992 in the Avon region of southwest England, exposure to IPV or maternal depression in childhood was associated with 24% to 68% higher risk of having severe depressive symptoms at age 18 years. The estimation of an individual developing depression in adolescence based solely on information about parental IPV or maternal depression is poor.

**Meaning:**

Prevention of IPV and maternal depression can improve children’s mental health at the population level; however, screening children for maternal depression and IPV to target interventions to prevent adolescent depression will not identify many children who might benefit and may unnecessarily target many others who will not develop depression.

## Teaser

This cohort study examines the extent to which experiencing parental intimate partner violence and/or maternal depression before age 12 years is associated with depression at age 18 years at the population and individual level.

## Introduction

Children’s exposure to intimate partner violence (IPV) between parents is defined as the “multiple experiences of children living in homes where an adult is using violent behaviour in a pattern of coercion against an intimate partner.”^1^ IPV appears to be bidirectionally associated with depression in the targets of such violence, typically mothers. For example, in a longitudinal study of Australian mothers, the risk of depression following IPV was 5-fold.^2^ In turn, there is evidence that depression renders a person more vulnerable to IPV; a meta-analysis^3^ of 41 studies found that the risk of lifetime partner violence against women with depressive disorders was nearly 3 times higher compared with women without depressive symptoms, and the risk was 4 times higher for those with anxiety disorders vs those without. Both parental IPV and maternal depression are associated with an elevated average risk of depression in children at the population or group level.^4,5^ However, it is unclear whether this association at the population level is also present at the individual level—namely, whether exposure to parental IPV and/or maternal depression differentiates between individuals who do and do not experience depression later. Phrased another way, if we were to screen for IPV and maternal depression would we have the potential to accurately target interventions at children at high risk of adolescent depression?

Previous research^6,7^ found that a count of a wider range of adverse childhood experiences, including exposure to violence and parental mental health problems, had very poor accuracy in estimating which children would experience a mental health problem. The studies^6,7^ showed that a person with multiple adversities had 8% to 15% higher probability of developing a mental health problem than a random individual. These findings warn against treating average associations present at the population level in a deterministic fashion at the individual level. In our study, we are specifically interested in parental IPV and maternal depression, because they are highly prevalent and are often detected at primary care visits. All clinical contacts, including general practitioner appointments or emergency department visits, present an opportunity to ask about potential mental health problems among children, which may help to identify vulnerable children early.^8^ Likewise, organizations tackling IPV can work closely with primary care and mental health services, as advocated by the World Health Organization.^9^ However, this targeted approach to preventive mental health interventions is most effective in identifying children at elevated risk if information about their exposure to parental IPV and/or mother’s depression accurately estimates those who will and will not develop depression.

We aimed to examine the extent to which exposure to parental IPV and/or maternal depression in childhood is associated with depression at age 18 years, both at the population and individual level. On the basis of previous studies^6,7^ of wider adverse childhood experiences, including parental IPV and maternal depression, we hypothesize that these exposures will be associated with adolescence depression at the population level but have a little estimating value at the individual level.

## Methods

### Data

This cohort study used data from the Avon Longitudinal Study of Parents and Children (ALSPAC), a population-based birth cohort from the Avon region of southwest England. ALSPAC initially recruited 14 541 pregnant mothers with estimated due dates between April 1991 and December 1992.^10,11,12^ After bolstering the initial sample with eligible participants who did not join the study originally, the total sample was 15 454 pregnancies resulting in 15 589 known fetuses, 14 901 of whom were alive at 1 year of age. Further details of the design of ALSPAC can be found elsewhere.^11,12^ Ethical approval was obtained from the ALSPAC Ethics and Law Committee and the Local Research Ethics Committee. Written informed consent was provided from all participants or their carers for each data collection. Reporting followed the Strengthening the Reporting of Observational Studies in Epidemiology (STROBE) reporting guideline. The study website contains details of all the data that is available through a fully searchable data dictionary and variable search tool.^13^

### Measures

#### Parental IPV and Maternal Depression

Parental (male-to-female) IPV included instances of the partner being physically or emotionally cruel to the mother at any time between child’s birth and 11 years, as self-reported via questionnaires by mothers. IPV was measured on 8 occasions when the children were aged approximately 1, 2, 3, 4, 5, 6, 9, and 11 years.

Maternal depression was defined as taking medications for depression or scoring above the established cutoff for depression on the Edinburgh Postnatal Depression Scale, equal to a score of 13 or higher, as reported via questionnaires by mothers. Maternal depression was measured on 8 occasions when the study children were approximately between the ages of 2 and 12 years (at approximately ages 2, 3, 4, 5, 6, 9, 10, and 12 years). We derived an indicator of being exposed to parental IPV or maternal depression between child’s birth and age 12 years, with 4 categories: (1) not exposed to either parental IPV or maternal depression, (2) exposed to IPV only, (3) exposed to maternal depression only, and (4) exposed to both IPV and maternal depression.

#### Adolescent Depressive Symptoms

Depressive symptoms were measured with the Short Mood and Feelings Questionnaire (SMFQ)^14^ and Clinical Interview Schedule–Revised (CIS-R),^15^ which were administered to study children at a clinic assessment at approximately age 18 years. The SMFQ includes 13 items that measure the presence of depressive symptoms in the last 2 weeks. The response scale for each question includes not true (scored 0), sometimes (scored 1), and true (scored 2), with the total score of summed items ranging from 0 to 26, where a higher score represents more severe symptoms. Following the recommendations of the validation study,^16^ we used the cutoff point of greater than 10 to indicate a potential diagnosis of depression.

The CIS-R was designed, on the basis of a clinical diagnostic tool, to be administered by non–clinically trained interviewers. The CIS-R asks questions about a range of symptoms and can be used to assign *International Statistical Classification of Diseases and Related Health Problems, Tenth Revision *diagnoses of depression and anxiety disorders, with the score ranging from 0 to 57 and a higher score indicating more and worse symptoms. In accordance with previous studies,^15^ a score of greater than 11 on the CIS-R indicates a case of depression. SMFQ and CIS-R were treated as 2 separate outcome variables.

#### Potential Confounding Factors

Potential confounding factors were chosen a priori on the basis of the previous literature and expert knowledge within the research team. They were defined as variables that were likely to be associated with the exposure and the outcome and were not on the causal pathway between these variables.^17^ They included child’s sex, family’s race, maternal marital status, maternal and paternal education, maternal and paternal occupational social class (Registrar General’s Social Class schema), financial difficulties, housing tenure, household crowding index, mother smoking during pregnancy, mother drinking alcohol during pregnancy, and mother’s age at birth, all measured between pregnancy and when the child was 1 year old. Participants’ self-identified race (dichotomized as White vs non-White, with no other response options available when the cohort was enrolled in 1991-1992) was assessed in this study because it is associated with structural differences in socioeconomic circumstances, which has implications for IPV and mental health. More details about potential confounding factors can be found in eTable 1 in Supplement 1.

### Statistical Analysis

#### Analytic Strategy

Data analysis was performed from February to March 2022. First, we examined the association between the exposure to parental IPV and/or maternal depression and adolescent depressive symptoms at age 18 years. Owing to the positive skewness of depressive symptoms, we used generalized linear model with a log link function (assuming Poisson distribution) and robust SEs. Differences in the risk of having a case of depression across levels of the exposures were expressed in relative terms as a risk ratio.

Second, we used receiver operating characteristic curve analysis to test whether being exposed to parental IPV and/or maternal depression discriminated between adolescents with and without depression. This analysis produces an area under the curve statistic, which indicates the probability of a child with parental IPV and/or maternal depression being more likely to report depression than a random individual. Values can range between 0.5 (chance) and 1.0 (perfect discrimination), with suggested grading as fail or very poor (0.5-0.6), poor (0.6-0.7), fair (0.7-0.8), good (0.8-0.9), and excellent (0.9-1.0).^18^

All analyses used sex-combined samples, because we did not hypothesize any differential effects due to sex. All analyses were conducted using Stata statistical software version 17 (StataCorp).^19^

#### Missing Data

The present study includes information from birth until the study children were 18 years old. After excluding those without information on either measure of depressive symptoms at age 18, the final sample comprised 5029 participants (eFigure in Supplement 1), which is 33.8% of the full ALSPAC sample (14 901 children alive at 1 year of age).

Information about IPV and/or maternal depression was missing for 53.3% of the sample, with measures of SMFQ and CIS-R missing for 10.7% and 9.4%, respectively, of the sample (eTable 2 in Supplement 1). Those with missing data tended to have higher depressive symptoms at age 18 years, have parental IPV, be more socioeconomically disadvantaged, be non-White, have more mental health problems in childhood, have a mother who smoked or drank during pregnancy, and have parents with mental health problems (eTable 3 in Supplement 1).

In the full available ALSPAC sample, the distribution of IPV and mother’s depression was highly comparable (Table 1 and eTable 4 in Supplement 1). Nonetheless, the study sample was socioeconomically more advantaged than the full sample, with, for instance, 19.3% of mothers (884 mothers) having a degree in the study sample compared with 12.9% (1598 mothers) in the full sample. There was also a lower proportion of non-White individuals in the study sample vs the full sample (192 participants [4.3%] vs 609 participants [5.1%]).

**Table 1.** Descriptive Information About the Studied Variables in the Study Sample

Variable (child’s age at time of measurement)	Participants, No (%) (N = 5029)
Outcome	
Short Mood and Feelings Questionnaire score >10 (age 18 y) (n = 4490)	
No	3518 (78.4)
Yes	972 (21.6)
Clinical Interview Schedule-Revised score >11 (age 18 y) (n = 4557)	
No	3856 (84.6)
Yes	701 (15.4)
Exposure to IPV or mother’s depression (ages 0-12 y) (n = 2349)	
No IPV and no mother’s depression (reference)	1437 (61.2)
IPV and no mother’s depression	211 (9.0)
No IPV and mother’s depression	479 (20.4)
IPV and mother’s depression	222 (9.5)
Confounding factors	
Child’s sex (n = 5029)	
Female	2862 (56.9)
Male	2167 (43.1)
Family’s race (n = 4507)^a^	
White	4315 (95.7)
Non-White	192 (4.3)
Maternal marital status (age 1 y) (n = 4638)	
Never married	632 (13.6)
Widowed, divorced, or separated	217 (4.7)
First marriage	3500 (75.5)
Second or third marriage	289 (6.2)
Maternal education (age 0 y) (n = 4574)	
Degree	884 (19.3)
A level	1281 (28.0)
CSE, vocational, or O level	2409 (52.7)
Paternal education (age 0 y) (n = 4464)	
Degree	1133 (25.4)
A level	1266 (28.4)
CSE, vocational, or O level	2065 (46.3)
Maternal social class (age 0 y) (n = 4093)	
I or II	1617 (39.5)
III nonmanual or III manual	1810 (44.2)
IV or V	666 (16.3)
Paternal social class (age 0 y) (n = 3677)	
I or II	1806 (49.1)
III nonmanual or III manual	1636 (44.5)
IV or V	235 (6.4)
Housing status (age 0 y) (n = 4598)	
Mortgaged or owned	3888 (84.6)
Not owned	710 (15.4)
Crowding index (age 1 y) (n = 4554)	
≤0.5	2349 (51.6)
>0.5-0.75	1375 (30.2)
>0.75-1	656 (14.4)
>1	174 (3.8)
Mother smoking during pregnancy (age 0 y) (n = 4640)	
No	3885 (83.7)
Yes	755 (16.3)
Mother drinking during pregnancy (age 0 y) (n = 4539)	
No	1314 (28.9)
Yes	3225 (71.1)
Financial difficulties (age 1 y), mean (SD), score [No.]^b^	2.38 (3.23) [4453]
Mother’s age at child’s birth, mean (SD), y [No.]^c^	29.75 (4.57) [4428]

Abbreviations: A level, advanced level; CSE, Certificate of Secondary Education; IPV, intimate partner violence; O level, ordinary level.

^a^ Race was dichotomized as White vs non-White, with no other response options available, when the cohort was enrolled in 1991-1992.

^b^ The score range was 0 to 15.

^c^ The age range was 16 to 45 years.

Missing data were replaced using multiple imputation by chained equations to minimize the bias due to nonresponse, resulting in 50 imputed samples.^20,21^ In line with recommendations, the imputation model included all analysis variables (ie, the exposure, outcomes, and confounding factors) ensuring that the relationship between the variables of interest was preserved.^20,21^ More details about the approach to missing data can be found in the eAppendix, eTable 2, and eTable 3 in Supplement 1.

## Results

### Characteristics of the Sample

Of the total sample of 5029 participants, 2862 (56.9%) were female and 2167 (43.1%) were male. The participants came mainly from families of White racial background (4315 participants [95.7%]).

Of 2349 children assessed for exposure to IPV or mother’s depression, 1437 (61.2%) had never experienced either, and 912 (38.8%) had experienced at least one (Table 1). In the imputed sample, 10.6% (95% CI, 9.2%-11.9%) of children experienced both IPV and mother’s depression between birth and age of 12 years, 21.5% (95% CI, 19.5%-23.4%) experienced mother’s depression only, 9.1% (95% CI, 7.9%-10.3%) experienced IPV only, and 58.9% (95% CI, 56.9%-60.9%) experienced neither IPV nor mother’s depression. The proportion of adolescent depression cases was 22.0% (95% CI, 20.8%-23.2%) according to the SMFQ and 15.6% (95% CI, 14.6%-16.7%) using the CIS-R. The correlation between both measures was moderate (ρ = 0.68). The proportion of cases was higher among those with either IPV or maternal depression than among those with neither of these experiences (Table 2). The proportion of cases in the group with both IPV and maternal depression was higher than in the groups with either exposure alone.

**Table 2.** Proportion of Depression Cases at Age 18 Years Across Different Levels of the Exposure, Based on Multiply Imputed Data^a^

Outcome	Depression cases, % (95% CI) (N = 5029)
Short Mood and Feelings Questionnaire score >10	
No IPV and no depression	18.2 (16.3-20.0)
IPV and no depression	23.8 (18.6-28.9)
No IPV and depression	25.8 (22.3-29.2)
IPV and depression	34.3 (28.8-39.7)
Overall	22.0 (20.8-23.2)
Clinical Interview Schedule–Revised score >11	
No IPV and no depression	12.1 (10.5-13.7)
IPV and no depression	17.9 (13.4-22.3)
No IPV and depression	20.1 (17.1-23.1)
IPV and depression	24.2 (19.1-29.4)
Overall	15.6 (14.6-16.7)

Abbreviation: IPV, intimate partner violence.

^a^ The tetrachoric correlation between being a case according to Clinical Interview Schedule–Revised and Short Mood and Feelings Questionnaire was 0.68.

### Associations at the Population Level

The associations between the exposure to IPV and/or maternal depression and adolescent depression were similar according to the SMFQ and CIS-R (Table 3). For brevity, we present results using SMFQ only in the text. In the confounding-adjusted model, experiencing IPV only was associated with 24% (risk ratio, 1.24; 95% CI, 0.97-1.59) higher risk of having depression, experiencing maternal depression only was associated with 35% (risk ratio, 1.35; 95% CI, 1.11-1.64) higher risk, and experiencing both IPV and maternal depression was associated with 68% (risk ratio, 1.68; 95% CI, 1.34-2.10) higher risk.

**Table 3.** Association Between IPV, Mother’s Depression, and Child’s Depression at the Population Level, Based on Multiply Imputed Data

Outcome (N = 5029)	Risk ratio (95% CI)	
	Unadjusted	Adjusted^a^
Short Mood and Feelings Questionnaire score >10		
No IPV and no mother’s depression	1 [Reference]	1 [Reference]
IPV and no mother’s depression	1.31 (1.02-1.67)	1.24 (0.97-1.59)
No IPV and mother’s depression	1.42 (1.17-1.71)	1.35 (1.11-1.64)
IPV and mother’s depression	1.89 (1.53-2.33)	1.68 (1.34-2.10)
Clinical Interview Schedule–Revised score >11		
No IPV and no mother’s depression	1 [Reference]	1 [Reference]
IPV and no mother’s depression	1.47 (1.10-1.97)	1.38 (1.03-1.84)
No IPV and mother’s depression	1.66 (1.34-2.07)	1.54 (1.24-1.92)
IPV and mother’s depression	2.00 (1.50-2.66)	1.69 (1.26-2.26)

Abbreviation: IPV, intimate partner violence.

^a^ Confounding (all measured between pregnancy and when the child was 1 year old) included child’s sex, family’s race, maternal partnership status, maternal and paternal education, maternal and paternal social class, financial difficulties, housing tenure, crowding index, mother smoking during pregnancy, mother drinking during pregnancy, and mother’s age at birth.

### Associations at the Individual Level

Of those young people with depression (SMFQ score >10) at age 18 years, 9.8% had a mother who experienced IPV, 25.1% had a mother with depression, 16.5% had a mother who experienced both depression and IPV, and 48.6% had a mother who experienced neither (exact numbers of participants are not available because these percentages are based on imputed data). Percentages were similar for young people with depression as reflected in CIS-R scores.

From the area under the curve analyses, we found that accuracy in estimating which children, on the basis of their exposure to IPV and/or maternal depression, had probable depression at age 18 years was poor. The area under the curve was 0.58 (95% CI, 0.55-0.60) for depression according to the SMFQ and 0.59 (95% CI, 0.55-0.62) according to the CIS-R (Figure). This indicates a 58% to 59% probability (ie, 8% to 9% above chance) that a random participant who had depression at age of 18 years had an experience of either or both IPV and maternal depression than a random participant who did not have depression.

**Figure.**
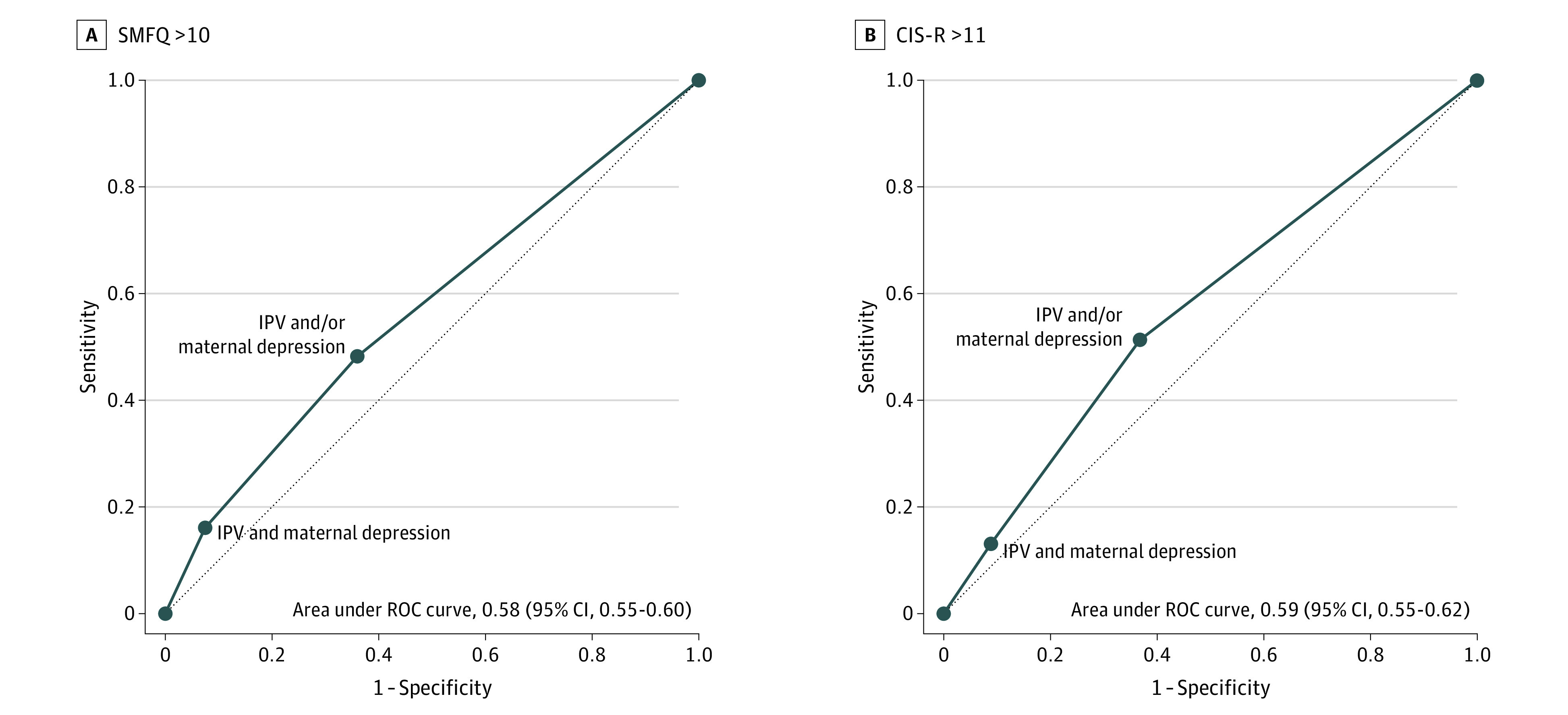
Accuracy of Estimations for Identifying Depression in Children on the Basis of Exposure to Intimate Partner Violence (IPV) and/or Mother’s Depression Data are shown for participants with Short Mood and Feelings Questionnaire (SMFQ) score greater than 10 (A) and Clinical Interview Schedule-Revised (CIS-R) score greater than 11 (B). The lines indicate receiver operating characteristic (ROC) curves with experiencing either IPV or mother’s depression as one discriminating threshold between those with or without depression, and with those having both exposures as another threshold. The dotted line signifies discrimination at chance level. The exact values of sensitivity and specificity can be found in eTable 5 in Supplement 1.

## Discussion

### Key Findings

One of the key strengths of this cohort study is the longitudinal design, spanning more than 18 years, and the use of prospectively collected information on all variables. Moreover, we accounted for a wide range of potentially confounding factors of the association, at the population level, between the exposure to parental IPV and/or mother’s depression and depression in adolescence. We found that experience of parental IPV or maternal depression in childhood was associated with a 24% to 35% higher average risk of having high enough levels of depressive symptoms at age 18 years to be indicative of a diagnosis of depression. Among those with experiences of both parental IPV and maternal depression, this risk increased to approximately 68%. However, the ability to estimate the risk of an individual developing depression in adolescence on the basis of only the information about their experience of parental IPV or mother’s depression was poor.

### Interpretation and Implications of the Findings

Parental IPV and maternal depression are highly prevalent, with more than 40% of our sample having experienced 1 or both exposures. On average, IPV and maternal depression were associated with a child’s depression. Hence, preventing these exposures from happening or reducing their impact on child’s depression may help to improve population mental health. For instance, *No Child Left Behind*, the recent report by Public Health England on vulnerable children,^22^ exemplifies the need to address family-level risk factors, highlighting domestic violence and mental illness within the household. However, these objectives are difficult to address because the evidence on effective strategies limiting the harmfulness of IPV and mother’s depression on child’s mental health is scarce.^22^

It is important to emphasize that most children from households experiencing IPV or depression will not develop mental health problems. This means that despite being associated with a higher risk of depression at the population level, IPV and maternal depression are not predictive of individual risk of depression. As found in our study, consistent with evidence on other childhood adversities,^6,7^ the accuracy of estimating whether children with the experience of parental IPV or/and maternal depression develop depression themselves is only 8% to 9% higher than that of a random child. It has been suggested that presentation to primary or secondary health care within families, for instance, because of injuries among mothers potentially indicating violence, can be used to identify vulnerable families for intervention.^23^ However, as emphasized previously by the Early Intervention Foundation^24^ and Public Health England,^22^ care needs to be taken not to treat this information in a deterministic fashion, assuming that the child will eventually develop depression. This could potentially result in stigmatization of children from vulnerable households and misallocation of resources.

### Limitations

This study has limitations that should be addressed. There is no guarantee that all potential confounding was considered. For instance, there may be genetic factors increasing the risk that family members will engage in violence and experience mental health problems.^25,26,27^ Nonetheless, childhood adversities such as IPV are typically considered to have social roots, for instance, due to socioeconomic disadvantage,^28^ which is also associated with mental health, and we controlled for a range of socioeconomic factors.

One of the key limitations of our study is related to attrition and nonresponse, both of which were found to be greater among non-White and socioeconomically disadvantaged populations, who typically report higher rates of IPV and mental health problems.^29,30^ We used multiple imputation to mitigate the potential impact and bias resulting from missing data.

Another limitation, as is typically the case with observational studies, was reliance on self-reports of IPV and depression both in mothers and their children. These can be underreported because of guilt or shame or fear of information being passed on, potentially resulting in underestimating the associations.^31^ Future research could attempt to replicate our findings in routinely collected data in health services, which would represent cases of IPV or depression severe enough to present to health care. There is a possibility that, although IPV is underrecorded in health care,^32^ the estimating accuracy of health care–recorded presentations of parental IPV and/or mother’s depression might be greater than in the population-based observational cohort studies of parental self-report, but this remains to be explored.

## Conclusions

The findings of this cohort study suggest that exposure to parental IPV or maternal depression before age 12 years affects 38.8% of children and, at the population level, is associated with depression in adolescence. However, the ability to estimate whether an individual develops depression in adolescence on the basis of only on the information from maternally reported parental IPV or depression is poor.

## Notes

Supplement 1. **eTable 1.** Details on Potential Confounding Factors **eFigure. **Sample Flow Diagram **eTable 2.** The Proportion of Missing Information in Each Variable in the Study Sample (n=5,029) **eTable 3.** Predictors of Having Missing Information on the Exposure or Either Outcome in the Study Sample (n=5,029) **eTable 4.** Descriptive Information About the Studied Variables: Full Available Sample **eAppendix.** Analytical Approach to Missing Data **eTable 5.** Predictive Accuracy for Identifying a Depression Case Based on the Exposure to Parental Intimate Partner Violence or/and Mother’s Depression

Supplement 2.
Data Sharing Statement
