# Supplementary material for: Association of Interparental Violence and Maternal Depression With Depression Among Adolescents at the Population and Individual Level
Source: JAMA Netw Open. 2023 Mar 1;6(3):e231175. doi: 10.1001/jamanetworkopen.2023.1175 (PMC9978945; doi:10.1001/jamanetworkopen.2023.1175)
Supplement: Supplement 1. — eTable 1. Details on Potential Confounding Factors eFigure. Sample Flow Diagram eTable 2. The Proportion of Missing Information in Each Variable in the Study Sample (n=5,029) eTable 3. Predictors of Having Missing Information on the Exposure or Either Outcome in the Study Sample (n=5,029) eTable 4. Descriptive Information About the Studied Variables: Full Available Sample eAppendix. Analytical Approach to Missing Data eTable 5. Predictive Accuracy for Identifying a Depression Case Based on the Exposure to Parental Intimate Partner Violence or/and Mother’s Depression [file jamanetwopen-e231175-s001.pdf]

Supplemental Online Content

Gondek D, Howe LD, Gilbert R, et al. Association of interparental violence and maternal depression with depression among adolescents at the population and individual level. *JAMA Netw Open*. 2023;6(3):e231175. doi:10.1001/jamanetworkopen.2023.1175

eTable 1. Details on Potential Confounding Factors

eFigure. Sample Flow Diagram

eTable 2. The Proportion of Missing Information in Each Variable in the Study Sample (n=5,029)

eTable 3. Predictors of Having Missing Information on the Exposure or Either Outcome in the Study Sample (n=5,029)

eTable 4. Descriptive Information About the Studied Variables: Full Available Sample

eAppendix. Analytical Approach to Missing Data

eTable 5. Predictive Accuracy for Identifying a Depression Case Based on the Exposure to Parental Intimate Partner Violence or/and Mother's Depression

This supplemental material has been provided by the authors to give readers additional information about their work.

© 2023 Gondek D et al. *JAMA Network Open.*

eTable 1. Details on Potential Confounding Factors	
Variable	
Child's sex	kz021
Maternal social class	Maternal and paternal social class were based on occupation during pregnancy (18 weeks pregnant). The original variables (ALSPAC: b_sc_m, pb_sc_p) were classified as: 1) I – professional, 2) II - managerial and technical, 3) IIINM - skilled non-manual, 4) IIIM - skilled manual, 5) IV - partly skilled, 6) V – unskilled. Due to low numbers of participants in certain categories, we reduced the number of categories to three: 1) I/II, 2) IIINM/IIIM, 3) IV/V.
Paternal social class	
Maternal education	Maternal and paternal education was self-reported (32 weeks pregnant). Using the original variables (ALSPAC: c645a, c666a), we derived three categories (due to low numbers in certain groups): 1) CSE/Vocational/O level, 2) A level, 3) Degree.
Paternal education	
Family's ethnicity	Family ethnicity was reported by mother (ALSAPC: c804, 32 weeks pregnant) as white or non-white.
Financial difficulties	Financial difficulties were reported by mother (ALSAPC: c525, 32 weeks pregnant). Mother was asked "how difficult at the moment do you find it to afford these items: 1) food, 2) clothing, 3) heating, 4) rent or mortgage, 5) things you will". The response options included: 0 – not difficult, 1 – slightly difficult, 2 – fairly difficult, 3 – very difficult. The responses were summed up, producing a score between 0 and 15, with a higher score indicating greater financial difficulties.
Mother smoking during pregnancy	Mothers were asked (ALSAPC: b665, 18 weeks pregnant) whether they smoked cigarettes, cigars or pipe during the first months of pregnancy). We binarized this variable as 'yes' (smoked any of these) or 'no' (did not smoke any of these).
Mother drinking during pregnancy	Mothers were asked about frequency of drinking alcohol in the first three months of pregnancy (ALSAPC: b721, 18 weeks pregnant) and after birth (ALSAPC: b721, after birth). Any indicator of drinking was classified as 'yes', not drinking at all was classified as 'no'.
Mother's age at birth	Age at birth was reported after self-reported (ALSAPC: c994, after birth).
Housing tenure	Home ownership status was self-reported by mother (ALSPAC: a006, 1 year old). We derived a variable indicating home being: 1) mortgaged/owned, 2) not owned.
Crowding index	Crowding index was derived by ALSPAC (ALSPAC: a551, 1-year-old) indicating the number of persons per room. It included four categories: 1) <= 0.5 persons per room, 2) >0.5 - 0.75 persons per room, 3) >0.75 – 1 persons per room, 4) > 1 persons per room.
Maternal partnership status	Maternal partnership status during pregnancy was self-reported by mother (ALSPAC: a525, 1 year old) mother. Due to low numbers in certain categories, we derive a variable with four categories: 1) Never married, 2) Widowed/divorced/separated, 3) 1st marriage, 4) 2nd/3rd marriage.

© 2023 Gondek D et al. *JAMA Network Open.*

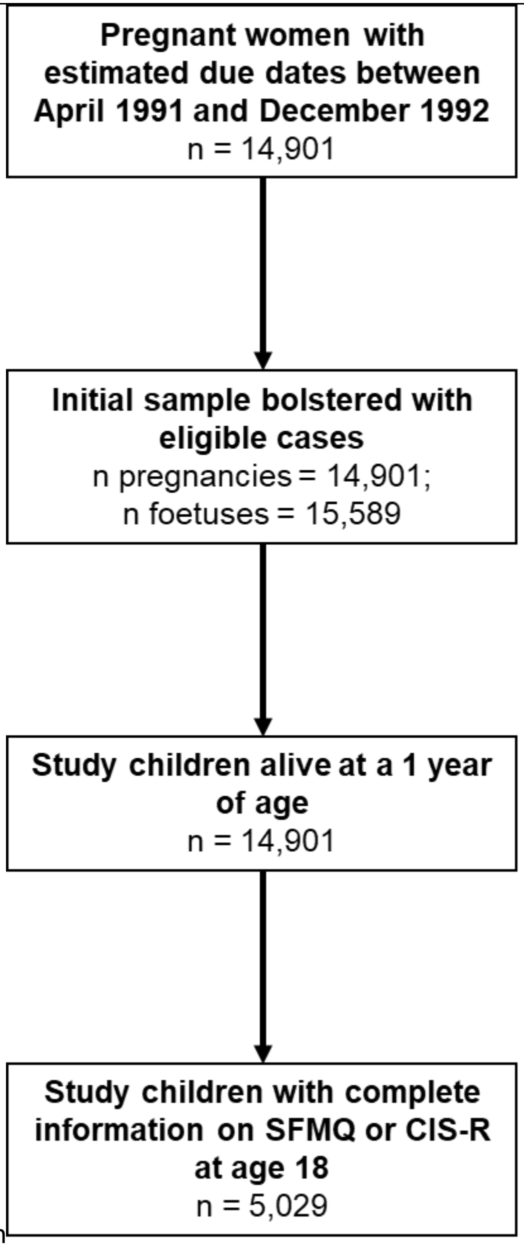

eFigure. Sample Flow Diagram

© 2023 Gondek D et al. *JAMA Network Open.*

eTable 2. The Proportion of Missing Information in Each Variable in the Study Sample (n=5,029)		
Variable (age in years when variables measured)	N missing	% missing
Outcomes		
Depressive symptoms (18) – SMFQ	539	10.7
Depressive symptoms (18) – CIS-R	472	9.4
Exposures		
IPV/maternal depression (0-12)	2,680	53.3
Confounding factors		
Child's sex	0	0.0
Maternal social class (0)	936	18.6
Paternal social class (0)	1,352	26.9
Maternal education (0)	455	9.1
Paternal education (0)	565	11.2
Family's ethnicity (0)	522	10.4
Financial difficulties (0)	576	11.5
Mother smoking during pregnancy (0)	389	7.7
Mother drinking during pregnancy (0)	490	9.7
Mother's age at birth (0)	601	12.0
Housing tenure (0)	431	8.6
Crowding index (0)	475	9.5
Maternal partnership status (0)	391	7.8
Auxiliary variables		
Birthweight	354	7.0
MFSQ = Short Mood and Feelings Questionnaire; CIS-R = Clinical Interview Schedule-Revised.		

© 2023 Gondek D et al. *JAMA Network Open.*

eTable 3. Predictors of Having Missing Information on the Exposure or Either Outcome in the Study Sample (n=5,029)			
Variables (age in years when variable measured)	Risk ratio	95% CI	
SMFQ (18) >10			
No (reference)	-	-	-
Yes	1.11	1.04	1.17
CIS-R (18) >11			
No (reference)	-	-	-
Yes	1.09	1.03	1.17
IPV/mother's depression (0-12)			
No IPV/no mother's depression (reference)	-	-	-
IPV/no mother's depression	0.89	0.63	1.25
No IPV/mother's depression	1.14	0.92	1.41
IPV/mother's depression	1.16	0.87	1.55
Maternal social class (0)			
I/II (reference)	-	-	-
III non-manual/III manual	1.05	0.99	1.11
IV/V	1.24	1.16	1.33
Maternal education (0)			
Degree (reference)	-	-	-
A level	1.16	1.06	1.26
CSE/Vocational/O level	1.32	1.22	1.42
Family's ethnicity			
White (reference)	-	-	-
Non-white	1.17	1.05	1.29
Mother smoking during pregnancy (0)			
No (reference)	-	-	-
Yes	1.27	1.20	1.33

Note. SMFQ = Short Mood and Feelings Questionnaire; CIS-R = Clinical Interview Schedule-Revised; CSE = Certificate of Secondary Education; O level = Ordinary level; A level = Advanced level; 95% CI = 95% confidence interval.

© 2023 Gondek D et al. *JAMA Network Open.*

eTable 4. Descriptive Information About the Studied Variables: Full Available Sample	
Variable (age in years when measured)	No (%)
Outcome	
SMFQ (18) >10	4,490
No	3,518 (78.4)
Yes	972 (21.6)
CIS-R (18) >11	4,557
No	3,856 (84.6)
Yes	701 (15.4)
Exposures	
IPV/mother's depression (0-12)	3,752
No IPV/no mother's depression (reference)	2,228 (59.4)
IPV/no mother's depression	332 (8.8)
No IPV/mother's depression	798 (21.3)
IPV/mother's depression	394 (10.5)
Confounding factors	
Child's sex	15,038
Female	7,348 (48.9)
Male	7,690 (51.1)
Family's ethnicity	12,136
White	11,523 (94.9)
Non-white	613 (5.1)
Maternal marital status (1)	13,544
Never married	2,595 (19.2)
Widowed/divorced/separated	816 (6.0)
1st marriage	9,252 (68.3)
2nd/3rd marriage	881 (6.5)
Maternal education (0)	12,478
Degree	1,608 (12.9)
A level	2,793 (22.4)
CSE/Vocational/O level	8,077 (64.7)
Paternal education (0)	11,996
Degree	2,178 (18.2)
A level	3,115 (26.0)
CSE/Vocational/O level	6,703 (55.9)
Maternal social class (0)	11,112
I/II	3,519 (31.7)
III non-manual/III manual	5,177 (46.6)
IV/V	2,416 (21.7)
Paternal social class (0)	9,517
I/II	3,918 (41.2)
III non-manual/III manual	4,760 (50.0)
IV/V	839 (8.8)
Housing tenure (0)	13,486
Mortgaged/owned	9,871 (73.2)
Not owned	3,615 (26.8)
Crowding index (1)	13,246
<= 0.5	5,484 (41.4)
>0.5 - 0.75	4,161 (31.4)
>0.75 – 1	2,679 (20.2)
> 1	922 (7.0)
Mother smoking during pregnancy (0)	13,344

© 2023 Gondek D et al. *JAMA Network Open.*

No	9,981 (74.8)
Yes	3,363 (25.2)
Mother drinking during pregnancy (0)	12,429
No	3,732 (30.0)
Yes	8,697 (70.0)
Financial difficulties (1) (n = 12,068), mean (SD)[1]	2.90 (3.53)
Mother's age during birth (0) (n = 11,983), mean (SD)[2]	28.64 (4.86)

Note. IPV = Interparental violence; SMFQ = Short Mood and Feelings Questionnaire; CIS-R = Clinical Interview Schedule-Revised; CSE = Certificate of Secondary Education; O level = Ordinary level; A level = Advanced level; 95% CI = 95% confidence interval; SD = standard deviation.

[1] Range = 0-15

[2] Range = 16-45

© 2023 Gondek D et al. *JAMA Network Open.*

eAppendix. Analytical Approach to Missing Data

The missing values were replaced with multiple imputation. The proportion of missing information in each variable is given in eTable 2. In line with the recommendations, we included all the variables from the analysis in the imputation model to preserve the relationship between the variables.[20,32]

The multiple imputation works under the missing at random (MAR) assumption.[33,34] The MAR mechanism implies that systematic differences between the missing and the observed values can be explained by observed data.[33] The missing-at-random assumption is largely untestable.[35] Hence, we also enriched the imputation model and further maximised the plausibility of the MAR assumption with birthweight as an auxiliary variable. This variable was not part of the substantive model of interest, but it was associated with missingness and with depressive symptoms. Including auxiliary variables can improve the accuracy of the MI and minimise non-random variation in the imputed values.[20] In addition, the imputation model was rich due to including covariates used in the analyses, such as socioeconomic indicators or mental health in childhood, which were predictive of both missingness (see eTable 3) and depressive symptoms.

The missing data were imputed using multiple imputation by chained equations (MICE), due to the non-monotone pattern of missing values, and due to its ability to accommodate various types of variables in the imputation model, including continuous and categorical ones. This approach uses a series of univariate conditional imputation models to impute missing data.[36] Continuous variables were imputed using predictive mean matching and categorical variables using logistic regressions. The predictive mean matching approach provides robust estimates if the normality assumption is in question,[37] this is particularly relevant to mental health outcomes as they tend to be skewed,[38] or when associations are non-linear.[37]

© 2023 Gondek D et al. *JAMA Network Open.*

eTable 5. Predictive Accuracy for Identifying a Depression Case Based on the Exposure to Parental Intimate Partner Violence or/and Mother's Depression

SMFQ – area under ROC curve = 0.58 (95% CI, 0.55, 0.61)					
Discriminative threshold	Sensitivity %	Specificity %	Correctly classified %	LR+	LR-
Either/both parental IPV and mother's depression	51.4	61.8	59.5	1.3	0.8
Both parental IPV and mother's depression	16.5	91.1	74.7	1.9	0.9
CIS-R – area under ROC curve = 0.59 (95% CI, 0.55, 0.63)					
Discriminative threshold	Sensitivity %	Specificity %	Correctly classified %	LR+	LR-
Either/both parental IPV and mother's depression	54.4	61.4	60.3	1.4	0.7
Both parental IPV and mother's depression	16.4	90.5	78.9	1.7	0.9

Note. SMFQ = Short Mood and Feelings Questionnaire; CIS-R = Clinical Interview Schedule-Revised; CSE = Certificate of Secondary Education; IPV = intimate partner violence; ROC = receiver operating characteristic; LR +/- = positive/negative likelihood ratio.
