# Supplementary material for: Association of Interparental Violence and Maternal Depression With Depression Among Adolescents at the Population and Individual Level
Source: JAMA Netw Open. 2023 Mar 1;6(3):e231175. doi: 10.1001/jamanetworkopen.2023.1175 (PMC9978945; doi:10.1001/jamanetworkopen.2023.1175)
Supplement: Supplement 2. — Data Sharing Statement [file jamanetwopen-e231175-s002.pdf]

Data Sharing Statement

Gondek. Association of Interparental Violence and Maternal Depression With Depression Among Adolescents at the Population and Individual Level. *JAMA Netw Open*. Published March 01, 2023. doi:10.1001/jamanetworkopen.2023.1175

Data
Data available: No

Additional Information
Explanation for why data not available: The ALSPAC dataset is available via a system of managed access. More information on access these data can be found at: http://www.bristol.ac.uk/alspac/researchers/access/
